# Effects of organic acid blends on the growth performance, intestinal morphology, microbiota, and serum lipid parameters of broiler chickens

**DOI:** 10.1016/j.psj.2024.104546

**Published:** 2024-11-10

**Authors:** Swapnali Waghmare, Mahesh Gupta, K.B. Bahiram, J.P. Korde, Rekha Bhat, Yashwant Datar, Pushpendra Rajora, M.M. Kadam, Megha Kaore, N.V. Kurkure

**Affiliations:** a Department of Veterinary Physiology, Nagpur Veterinary College, Maharashtra Animal & Fishery Sciences University, Nagpur, 440006, India; b Mankind Pharma Limited, 208, Okhla Phase 3 Rd, Okhla Phase III, Okhla Industrial Estate, New Delhi, Delhi 110020 India; c Department of Poultry Science, Nagpur Veterinary College, Maharashtra Animal & Fishery Sciences University, Nagpur, 440006, India; d Department of Veterinary Pathology, Maharashtra Animal & Fishery Sciences University, Nagpur, 440006, India

**Keywords:** Organic acids, Acidapure, Broiler, Growth performance, Gut morphology

## Abstract

Organic acids have emerged as promising alternatives to antibiotic growth promoters in poultry production. The present study was conducted to determine the effects of organic acids blends v.i.z. Acidapure liquid and Acidapure powder supplementation on the growth performance, gut health, gut microbiota, and serum lipid profile of broiler chickens. A total of 120-day-old chicks with similar live body weights were randomly divided into four groups. Each group was further divided into 3 replicates, and each further divided into three replicates of ten bird. The birds in Group 1 (T1) were fed a basal diet supplemented with plain drinking water, those in Group 2 (T2) received basal feed supplemented with Acidapure powder (1 kg/MT feed) and plain drinking water, those in Group 3 (T3) received basal feed supplemented with Acidapure liquid in the drinking water (0.2 ml/l water), and those in Group 4 (T4) received basal feed supplemented with Acidapure powder (1 kg/MT feed) and Acidapure liquid in the drinking water (0.2 ml/l water). Acidapure powder and Acidapure liquid were added to the feed and water of the broilers from 0–42 days of life. The results showed that compared with the control (T1), supplementation with Acidapure powder and liquid in broiler chickens for 42 days increased (P < 0.05) ABW and ADG and reduced FCR in the treatment groups (T2, T3 and T4). At d 21 and 42, all forms of Acidapure supplement increased the VH and CD in the jejunum and ileum and reduced the pH of the ileum. Compared with the control (T1), the combination of Acidapure powder and liquid (T4) increased the gene expression of the tight junction proteins *Claudin-1* and *Zona Occludense 1 (ZO-1)*. Compared with the control, Acidapure supplementation reduced the cecal coliform count and total viable count (TVC) and decreased the serum cholesterol and triglyceride levels. In conclusion, Acidapure, as a blend of organic acids, effectively enhances the growth performance and gut health of broilers, making it a viable and safe alternative to traditional antimicrobial growth promoters.

## Introduction

Antibiotics are routinely administered to food-producing animals in many regions of the world to accelerate their growth and prevent sickness. In the chicken industry, antibiotics are primarily used for prophylaxis, treatment, and growth promotion. Given the rising demand, this tendency regarding animal-sourced protein is likely to continue. The therapeutic use of antibiotics focuses on treating bacterial disease, and subtherapeutic levels are often used to promote growth and improve feed conversion ratios. This practice is further driven by the increasing global demand for animal-sourced proteins. However, this widespread and continuous use of antibiotics has contributed to the rise of antimicrobial resistance (AMR) in pathogenic microorganisms, which poses a significant risk to both animal and human health. Moreover, the presence of antibiotic residues in poultry products has raised further concerns about food safety and public health (Gadde et al. 2018; Mehdi et al., 2018).

The search for alternatives to in-feed antibiotics in the poultry industry has accelerated because of the European Union's ban on antibiotic use in animal production in 2006, followed by restrictions in many countries. These substitutes include immune stimulants, medicinal plants, prebiotics, probiotics, enzymes, and organic acidifiers. The majority of these alternatives influence microflora directly or indirectly. Among these, organic acids, also known as acidifiers, have gained significant attention due to their ability to maintain gastrointestinal health and improve nutrient absorption (Yang et al., 2018).

Organic acids are naturally occurring compounds found in various feeds or produced during an animal's metabolism. They are frequently utilized to acidify poultry feed, thus lowering the pH of both the feed and the gastrointestinal tract. This reduction in pH inhibits harmful bacterial growth while simultaneously promoting the digestion of proteins and the absorption of key minerals such as calcium, phosphorus, magnesium, and zinc (Khan et al., 2022). Studies have shown that the use of organic acids, such as citric, fumaric, lactic, and formic acids, in poultry diets can improve growth performance, enhance mucosal immunity, reduce pathogenic colonization, and decrease the production of harmful bacterial metabolites. Organic acids are also effective in poultry feed as anti-Salmonella agents due to their potent bacteriostatic properties (Mani-López et al., 2012). Additionally, feed acidifiers lower the microbial population in the gut, reducing nutrient competition between microbes and the host animal. This results in improved feed efficiency, enhanced growth, and better overall health in broilers (Dibner & Buttin, 2002). Moreover, acidifiers can prevent mold growth in feed, ensuring that the feed retains its nutritional value over time.

Organic acids in the short-chain fatty acid (SCFA) category have functional groups like carboxylic, hydroxyl, or double bonds. SCFAs’ antimicrobial effect in the gut depends on molecular size, dissociation constant (pKa), gut pH, and the microorganism involved (Mroz et al., 2006). Lipophilic undissociated SCFAs can penetrate bacterial membranes, where they dissociate, releasing protons (H+) that inhibit ATP synthesis and disrupt metabolism (Mroz et al., 2006). Accumulated acid anions further damage bacterial cell structures (Russell, 1992). However, some acid-tolerant, Gram-positive beneficial bacteria like *Lactobacillus* and *Bifidobacterium* can survive by neutralizing acid anions (Russell & Diez-Gonzalez, 1998). Organic acids thus promote a balanced gut environment, selectively inhibiting pathogens without harming beneficial microbes. Salt forms of these acids (e.g., sodium, potassium, and calcium) are preferred for their lower odor, reduced corrosiveness, and higher solubility.

The nutrients are absorbed in the small intestine; hence, it is a crucial part of the digestive system (Lin et al., 1999). Improving the structural morphology of the gut increases the digestive and absorptive functions of the intestine by expanding the absorptive surface area (Choct, 2009; Yadav et al., 2022). Hence, intestinal histology in general and the jejunum in particular are vital for intestinal function. The inhibition of intestinal bacteria from competing with the host for available nutrients and reducing possible toxic bacterial metabolites in the gut are beneficial effects of dietary acidification (Guo et al., 2022). Multiple organic acids (butyric, fumaric, and lactic acid) have been studied for their effects on chickens at various levels by Adil et al. (2010). The findings showed that acid had a beneficial effect on bird body weight gain and the feed conversion ratio and increased the villus height in the small intestine, irrespective of the type and acid concentration. Blends of acidifiers and sodium butyrate have also been shown to be antioxidants that prevent free radical damage caused by heat stress (Awaad et al., 2018). Vinolya et al. (2021) observed that supplementing broiler diets with a blend of short-chain organic acids—fumaric, lactic, benzoic, propionic, and formic acids—and essential oils improved broiler performance and economic returns compared to inorganic and medium-chain organic acids, especially under challenging dietary conditions with animal protein sources.

Despite the promising results, significant gaps remain in our understanding of the full potential of organic acids as antibiotic replacements in poultry feed. One key area of uncertainty is the variability in outcomes reported by different studies, which makes it difficult to generalize the effects of organic acids on broiler performance. Factors such as the type and concentration of the acid, the blend used, the age and genetic background of the birds, and environmental conditions all influence the effectiveness of organic acid supplementation. Furthermore, the long-term impact of organic acids on poultry health, including potential impacts on the gut microbiome, immune function, and resistance to disease, requires more investigation. Given the increasing demand for antibiotic-free poultry products, exploring the potential of various organic acids and their blends is critical.

The present study aims to address these knowledge gaps by evaluating the effects of newly developed organic acid blends, Acidapure powder and Acidapure liquid, on the growth performance, gut health, microbiota, and serum lipid profiles of broiler chickens. By investigating the efficacy of these novel acidifiers, this research seeks to contribute to the development of sustainable and effective alternatives to antibiotics in poultry production. Understanding the interactions between organic acids, gut health, and overall bird performance will be critical for advancing antibiotic-free poultry farming practices

## Materials and methods

### Experimental design, birds, and management

The experiment was approved by the Institutional Animal Ethics Committee of Nagpur Veterinary College (Approval Number NVC/IAEC/23/2022). A total of 120, day-old, Vencobb 430 commercial broiler chicks purchased from a local hatchery were randomly allocated into 4 treatment groups: T1: fed with corn‒soybean meal basal diets (control group); T2: basal diet supplied with organic acid blend Acidapure Powder @ 1Kg/MT of feed; T3: basal diet supplemented with organic acids and essential oil blend Acidapure Liquid in drinking water @ 0.2 ml/l of water daily; and T4: basal diet supplied in combination with Acidapure Powder @ 1kg/MT of feed and Acidapure Liquid @ 0.2 ml/l of water daily in drinking water. Each treatment included 3 replicates of 10 chicks/cage. The organic acid blends were provided by Mankind Pharma, New Delhi, India. The Acidapure powder consisted of a mixture of acetic acid, formic acid, propionic acid, benzoic acid, sorbic acid, butyric acid, and oxine copper. The ingredients of Acidapure liquid were lactic acid, formic acid, citric acid, sodium citrate, butyric acid, oxine and copper.

The birds were fed a prestarter (d 0-7), starter (d 8-21), or finisher diet (d 22−42). The basal diets (Table 1) were formulated to meet the Bureau of Indian Standards (2007). The composition of the basal diets and proximate analysis results are depicted in Table 1. The trial was conducted for 42 days. All chicks were vaccinated for Newcastle disease on the 7th day and 21st day of age. Ad libitum access to feed and water was provided. All birds remained healthy throughout the trial.

**Table 1:** Ingredient composition of basal diet and nutrient levels.

**Feed Ingredients**	**Pre-Starter** **(0-7 days)**	**Starter** **(8-21 days)**	**Finisher** **(22-42 days)**
Maize	57.50	59.00	61.00
Soybean meal	36.40	33.45	30.00
Vegetable oil	1.75	3.20	4.50
Monocalcium phosphate	1.50	1.40	1.50
Limestone powder	1.40	1.50	1.65
Salt	0.30	0.30	0.30
Trace Min. Premix	0.05	0.05	0.05
Vitamin Premix	0.05	0.05	0.05
DL-Methionine	0.30	0.35	0.30
L-Lysine	0.30	0.25	0.25
L-Threonine	0.15	0.15	0.10
Choline Chloride	0.10	0.10	0.10
Toxin binder	0.15	0.15	0.15
Coccidiostat	0.05	0.05	0.05
Total	100	100	100
**Nutrient levels (%)**			
Dry matter	88.9	89.3	90.2
Crude protein	23.4	21.8	20.1
Ether extract	4.09	5.20	6.30
Crude fibre	3.68	3.50	3.32
N.F. E	62.61	63.18	63.79
Total ash	6.22	6.32	6.49
ME (Kcal/kg)	3154	3252	3280

### Growth performance

The birds and feed were weighed by pen at weekly intervals for determination of growth performance, which included average body weight (ABW), average daily gain (ADG), average daily feed intake (ADFI), and the feed conversion ratio (FCR). Mortalities, if any, were noted daily, and postmortem weight was recorded for the calculation of mortality and body weight gain. The FCR was calculated by dividing weight gain by feed intake.

### Intestinal morphology and gut pH

On days 21 and 42, six birds per treatment (2 birds per replicate) were sacrificed, and intestinal tissues were collected to analyze intestinal morphology. Before that, birds were fasted for 12 hours. The middle segments of the duodenum, jejunum, and ileum (approximately 2 cm) were collected and fixed in 10% neutral buffered formalin. After being washed, dehydrated, and clarified, the samples were embedded in paraffin. Serial sections with a thickness of 5 µm were placed on glass slides for staining (H & E). The villus height (VH) and crypt depth (CD) were measured via the CARL ZEISS ZEN image processing and analysis system (CARL ZEISS Germany). The ratio of villus height/crypt depth (VH/CR) was calculated. The pH of the gut contents of the duodenum, jejunum and ileum was measured on days 21 and 42.

### Real-time quantitative PCR

On day 42, the jejunum mucosa of six birds from each treatment group was collected to analyze the gene expression of the tight junction protein genes v.i.z. *Occludin, Claudin-1,* and *Zona occludense 1* (*ZO-1)*. The total RNA of the jejunum mucosa samples was extracted with TRIzol reagent (Thermo Fisher Scientific, Wilmington, DE), and the purity and concentration of the total RNA were determined with a BioSpectrometer. Then, 1 mg of RNA from each sample was reverse transcribed into cDNA via the GoScript reverse transcription system (Promega, USA) following the manufacturer's instructions. The GoTaq qPCR SYBR Master Mix (Promega, USA) was used to carry out real-time quantitative polymerase chain reaction (qRT‒PCR) on an Insta Q 96 real-time PCR Design & Analysis system (Himedia, India). The sequences of primers used in this study are shown in Table 2. The relative mRNA expression levels were normalized to those of avian β-actin by 2−∆∆Ct method (Livak and Schmittgen, 2001).

**Table 2:** Primer sequences used for qRT-PCR.

Gene	Sequences 5′-3′	Efficiency (%)	Amplicon length (bp)	Reference
*Occludin*	For: ACGGCAGCACCTACCTCAA Rev: GGGCGAAGAAGCAGATGAG	102	123	Wu *et al*., 2021
*Claudin-1*	For: CATACTCCTGGGTCTGGTTGGT Rev: GACAGCCATCCGCATCTTCT	98.5	100	Wu *et al*., 2021
*ZO-1*	For: CTTCAGGTGTTTCTCTTCCTC Rev: CTGTGGTTTCATGGCTGGATC	104.5	131	Wu *et al.,* 2021
*ACTB*	For: GAGAAATTGTGCGTGACATCA Rev: CCTGAACCTCTCATTGCCA	108.2	152	Wu *et al*., 2021

### Microbiological analyses of cecal contents

Cecal contents from all sacrificed birds at both time points were analyzed for microbial populations using a conventional method (Shokryazdan et al., 2017). Briefly, one g of cecal content was suspended in 9 ml of phosphate-buffered saline and vortexed for 1 min. Samples were serially diluted in sterile distilled water, and 100 μl of 10^−4^ to 10^−6^ dilutions were streaked on appropriate selective media for enumeration of different groups of bacteria. Nutrient agar was used for total aerobic counts, and Eosin Methylene Blue agar was used for *E. coli* counts. After incubation under appropriate conditions for each group of bacteria (48 h at 39°C under aerobic conditions), colonies on the plates were counted, and the microbial population was expressed as log_10_ CFU g^-1^ cecal content.

### Humoral immune response against Newcastle disease

The humoral immune response was judged by estimating the hemagglutination inhibition titer against Newcastle disease virus (NDV) at the 21st and 42nd days of age. For this purpose, blood was collected from two birds per replicate. Blood samples were allowed to settle at room temperature for 1 h and then centrifuged at 3000 × g for 10 min. The serum was transferred into two vials and stored at -20°C until use. A hemagglutination inhibition test (HI) was subsequently performed to determine the titer against NDV. The results were expressed as log_2_ values of the highest serial dilution showing complete inhibition of the 4HA unit of the test antigen.

### Serum lipid assay

The serum stored in another vial was analyzed for total cholesterol, high-density lipoprotein (HDL)-cholesterol, low-density lipoprotein (LDL)-cholesterol, and triglycerides using the kits from Delta Lab (New Delhi, India). The determination was based on the enzyme colorimetric method.

### Statistical analysis

The data collected in this study were statistically analyzed via SPSS 21 software. One-way analysis of variance (ANOVA) was used to analyze the data. Means were compared and separated using the Tukey post hoc test. The significance level was at 5%. The results were expressed as the means and standard error of the mean (SEM).

## Results

### Effects of Acidapure powder and Acidapure mixture on the growth performance of broilers

The effects of the addition of the Acidapure powder, Acidapure liquid, and their combination on the growth performance of broilers are summarized in Table 3. There was 2% mortality in birds during the experimental period. On day 21, the average body weight (ABW) of birds who received Acidapure (liquid or powder) was significantly (P < 0.05) greater than that of control birds (T1). The ABW of the birds that received Acidapure liquid (T3) or a combination of liquid and powder (T4) was greater than that of the birds that received Acidapure powder in the feed (T2). On day 42nd, the birds receiving Acidapure in powder or liquid form have higher weight than the control birds. The ADG during the entire experimental period (days 1-42) was significantly greater (P < 0.05) in the birds receiving Acidapure powder or liquid than in the control birds. Compared with the control (T1), the combination of Acidapure powder and liquid (T4) reduced the ADFI; however, the ADFI was similar in the T1, T2 and T3 groups. Acidapure supplementation in any form (T2, T3, or T4) significantly reduced the FCR from days 1-21 and from days 1-42. In general, Acidapure supplementation improved the performance of broilers.

**Table 3:** Effect of supplementation in Acidapure powder & liquid on growth performance of broilers.

**Parameters**	**Dietary Treatments**				SEM	P Value
	T1	T2	T3	T4		
**ABW (g/bird)**						
D 1	45.13±0.09	44.23±0.47	46.19±0.12	45.12±0.28	0.16	0.09
D 21	811.89^c^±20.34	850.34^b^±15.56	890.23^a^±25.35	905.22^a^±30.75	22.52	<0.05
D 42	2282.12^b^±58.75	2398.67^a^±63.12	2410.12^a^±68.49	2425.77^a^±85.53	73.59	<0.05
**ADG (g/d/bird)**						
D 1-21	39.09^b^±1.15	40.49^b^±1.65	42.39^ab^±1.31	43.10^a^±1.80	1.55	<0.05
D 22-42	66.52±3.06	73.49±3.38	72.37±5.41	72.40±5.30	4.53	0.12
D 1-42	52.33^b^±2.08	57.11^a^±2.11	57.38^a^±1.96	58.75^a^±2.25	2.15	<0.05
**ADFI (g/d/bird)**						
D -21	53.84^b^±1.80	48.61^a^±2.12	47.95^a^±2.27	47.38^a^±2.07	2.13	<0.05
D 22-42	143.23^a^±3.44	137.85^ab^±4.23	134.61^b^±3.50	131.19^b^±4.12	3.83	<0.05
D 1-42	96.03^a^ ±2.65	93.23^a^±3.12	91.28^a^ ±2.84	89.28^b^±2.31	3.65	<0.05
**Average C-FCR**						
D 1-21	1.38^a^±0.04	1.29^b^±0.02	1.28^b^±0.03	1.25^b^±0.03	0.04	<0.05
D 1-42	1.75^a^±0.02	1.66^b^±0.01	1.64^b^±0.02	1.62^b^±0.03	0.09	<0.01

### Effects of Acidapure powder and Acidapure liquid on gastrointestinal tract contents pH and gut morphology

As shown in Table 3, no significant differences were noted among the groups in terms of the pH values of the contents of the duodenum and jejunum. However, the addition of either the Acidapure powder or the Acidapure mixture tended to decrease the pH. Compared with that of the control group, the pH of the ileum was significantly lower with the addition of Acidapure powder and liquid (P < 0.05).

The effects of Acidapure supplementation on the intestinal morphology of broilers are shown in Table 4. On day 21st and day 42nd, the groups receiving the Acidapure powder or liquid or their combination had higher (P < 0.05) VH and CD of jejunum and ileum in comparison to the control group. VH and CD were comparable among the Acidapure powder (T2), Acidapure liquid (T3), and their combination groups (T4). The VH/CD ratio was significantly greater in acidapure-supplemented birds than in control birds on day 42. The present results indicated that Acidapure supplementation promoted the development of the small intestine.

**Table 4:** Effect of supplementation in Acidapure powder and liquid on gut pH and gut morphology of broilers.

**Parameters**		**Dietary Treatments**				SEM	P Value
		T1	T2	T3	T4		
**Gut pH**							
D 21	Duodenum	5.30±0.28	5.23±0.20	5.20±0.05	5.13±0.03	0.12	0.09
	Jejunum	6.75±0.16	6.56±0.31	6.47±0.09	6.23±0.00	0.10	0.08
	Ileum	7.48^a^±0.12	6.85^b^±0.28	6.05^b^±0.43	6.16^b^±0.14	0.17	<0.05
D 42	Duodenum	5.80±0.06	5.03±0.19	5.11±0.12	5.16±0.18	0.08	0.07
	Jejunum	6.90±0.21	6.62±0.02	6.55±0.09	6.52±0.10	0.07	0.12
	Ileum	7.52^a^±0.06	6.74^b^±0.28	6.57^b^±0.36	6.65^b^±0.26	0.22	<0.05
**Gut morphology**							
D 21 VH (µm)	Jejunum	805.71^b^±21.58	961.00^a^±18.01	954.33^a^±22.89	985.00^a^±25.19	22.61	<0.05
	Ileum	627.64^b^±23.52	757.70^a^±14.66	749.67^a^±18.84	793.67^a^±28.15	22.26	<0.05
D 42 VH (µm)	Jejunum	993.21^b^±24.51	1162.47^a^±39.13	1185.52^a^±34.6	1232.67^a^±33.83	41.71	<0.01
	Ileum	738.56^b^±20.73	877.29^a^±16.10	876.02^a^±10.58	902.00^a^±16.66	14.63	<0.01
D 21 CD (µm)	Jejunum	146.29^b^±3.99	155.20^a^±3.61	163.00^a^±4.04	170.92^a^±3.48	8.75	<0.05
	Ileum	144.04^c^±5.38	155.67^b^±3.48	158.12^b^±6.45	168.00^a^±3.51	8.26	<0.05
D 42 CD (µm)	Jejunum	173.43^b^±3.49	189.96^a^±5.23	192.00^a^±7.00	200.29^a^±8.30	7.02	<0.05
	Ileum	153.18^b^±10.57	167.66^a^±6.94	165.67^a^±6.12	178.96^a^±5.27	8.20	<0.05
D 21 VH/CD	Jejunum	5.59±0.11	6.20±0.14	5.85±0.14	5.79±0.12	0.11	0.08
	Ileum	4.67±0.08	4.88±0.12	4.74±0.06	4.72±0.18	0.10	0.10
D 42 VH/CD	Jejunum	5.73^a^±0.10	6.14^b^±0.14	6.17^b^±0.16	6.16^b^±0.18	0.16	<0.05
	Ileum	4.82^a^±0.06	5.25^b^±0.15	5.30^b^±0.18	5.06^b^±0.08	0.11	<0.05

### Effect of Acidapure supplementation on the gut microbial count

The effects of Acidapure supplementation on the cecal microbiota (total viable count and coliform count) are presented in Table 5. On the 21st day, no significant differences were observed in the total viable count or coliform count among all the treatment groups. On day 42, a significant reduction in coliform count was recorded in the T2, T3, and T4 treatment groups (Acidapure supplemented) compared with the T1 (control) group of birds. Additionally, the TVC was reduced significantly (P < 0.05) on day 42 by Acidapure supplementation. The TVC was lower in the combined Acidapure powder and Acidapure liquid-supplemented group (T4) than in the control (T1) group.

**Table 5:** Effect of supplementation in Acidapure powder and liquid on gut microbial count of broilers.

**Microbial count** (log_10_ CFU g^-1^)		**Dietary Treatments**				SEM	P Value
		T1	T2	T3	T4		
D 21	**TVC**	6.27±0.26	5.88±0.21	5.38±0.26	5.22±0.29	0.17	0.14
	**E. coli**	4.41±0.12	4.63 ±0.18	4.35±0.20	4.25±0.16	0.13	0.09
D 42	**TVC**	7.33^a^±0.25	6.72^b^±0.23	6.45^b^±0.20	6.28^bc^±0.25	0.23	<0.05
	**E. coli**	5.72^a^±0.12	4.47^b^±0.16	4.45^b^±0.14	4.39^b^±0.09	0.13	<0.05

### Effect of Acidapure supplementation on the mRNA expression of tight junction proteins

The mRNA expression levels of tight junction protein genes (*Claudin-1, Occludin*, and *ZO-1*) in the chicken jejunum mucosa on day 42 are shown in Table 6. *The expression of occludin* did not differ among the different treatment groups. The expression of *Claudin-1* and *ZO-1* was significantly upregulated in the combined Acidapure powder and liquid-supplemented group (T4) compared with the control (T1). In the Acidapure powder (T2)- and Acidapure liquid (T3)-supplemented groups, the mRNA expression of claudin and ZO-1 tended to increase; however, it did not significantly differ from that in the control group of birds (T1).

**Table 6:** Effect of supplementation in Acidapure powder and liquid on Gene expression of broilers.

**Dietary Treatments**	Expression of genes		
	*Claudin-1*	*Occludin*	*ZO-1*
T1	1.01^b^±0.09	1.00±0.15	1.07^b^±0.06
T2	1.21^b^±0.13	1.12±0.09	1.20^b^±0.12
T3	1.18±0.10	1.20±0.13	1.24^b^±0.13
T4	1.50^a^±0.12	1.23±0.15	1.69^a^±0.12
SEM	0.10	0.13	0.11
P Value	<0.05	0.09	<0.05

Values indicate mean ± S.E. Mean values with different alphabets as superscripts differ significantly (P≤0.05).

### Effect of Acidapure supplementation on the serum lipid profile

The results of the parameters of the serum lipid profile v.i.z. total cholesterol, HDL cholesterol, LDL cholesterol, and serum triglycerides are presented in Table 7. Compared with the control (T1), the combination of the Acidapure powder and Acidapure mixture (T4) significantly reduced the total cholesterol level. Acidapure supplementation had no effect on HDL or LDL; however, acidapure supplementation in powder, liquid, or a combination significantly (P < 0.05) reduced the serum triglyceride level.

**Table 7:** Effect of supplementation in Acidapure powder and liquid on Lipid profile of broilers.

**Dietary Treatments**	Serum lipid parameters			
	Total Cholesterol (mg/dl)	HDL (mg/dl)	LDL (mg/dl)	Triglycerides (mg/dl)
T1	139.13^a^±2.67	50.79±1.06	61.10±2.09	96.50^a^±1.69
T2	133.26 ^ab^ ±3.09	54.26±2.91	59.04±1.55	86.76^b^±2.63
T3	135.57^ab^±3.57	53.53±1.53	55.90±2.12	82.93^c^±1.40
T4	131.79^b^±2.17	52.34±1.64	53.53±2.15	88.79^b^±2.87
SEM	3.33	2.18	1.98	2.07
P Value	<0.05	0.07	0.13	<0.05

### Effect of Acidapure supplementation on the humoral immune response against Newcastle disease virus

The humoral immune response against Newcastle disease virus in terms of the HI titer is presented in Table 8. There was no effect of Acidapure on the humoral immune response against NDV in broilers on days 21st and 42nd of the experimental period.

**Table 8:** Effect of supplementation in Acidapure powder and liquid on HI titre against Newcastle Disease Virus in different groups of broilers.

Dietary Treatments	HI titre (D 21)	HI titre (D 42)
T1	2.10±0.10	2.21±0.10
T2	2.32±0.04	2.32±0.02
T3	2.21±0.10	2.21±0.10
T4	1.96±0.21	2.21±0.10
SEM	0.06	0.04
P Value	0.33	0.80

## Discussion

As antimicrobial growth promoters are forbidden in many production systems, organic acids have been widely used in the poultry industry to improve the health and productivity of broilers. Among organic acids, fumaric, formic, lactic, butyrate, propionic, and citric acids, as well as their salts have been extensively investigated and used for improving broiler productivity with varied degrees of efficacy (Leeson et al., 2005; Kopecký et al., 2012; Ma et al., 2021; Gao et al., 2021; Sizmaz et al., 2022). An acidifier is typically a blend of several organic acids. In the present study, we investigated the effects of acidifier blends, i.e., Acidapure powder (containing acetic acid, formic acid, propionic acid, benzoic acid, sorbic acid, butyric acid, and oxine copper), Acidapure liquid (containing lactic acid, formic acid, citric acid, sodium citrate, butyric acid, oxine copper) and a combination of Acidapure powder in feed and Acidapure liquid in water.

Our data revealed that the addition of Acidapure powder @ 1 Kg/MT in the diet or Acidapure liquid @ 0.2 ml/l in drinking water or both increased ABW and ADG. No significant differences were observed in the ADFI with either Acidapure powder or liquid supplementation; however, the combination reduced daily feed intake. Acidapure supplementation also reduced FCR. These results show that Acidapure improves the growth performance of broilers. Similar to our findings, several previous studies have reported that the addition of organic acid mixtures could increase ADG or improve FCR in broiler chickens reared under either nonchallenged (Chowdhury et al., 2009; Adil et al., 2010; Hashemi et al., 2012; Gao et al., 2021; Huang et al., 2024) or challenged conditions (Mustafa et al., 2021). Organic acid (citric acid) also improved the growth performance of geese (Xue et al., 2023). In another study, Vinolya et al. (2021) noted that supplementation of short-chain organic acid blend containing fumaric, lactic, benzoic, propionic, formic acid and organic acid with essential oil enhances the performance of broilers with better economic return under challenged dietary conditions with animal protein sources. In contrast to the above mentioned studies, some studies have reported that dietary supplementation with organic acids has no significant or even negative effects on growth performance (Kopecký et al., 2012; Adewole et al., 2021). The differences in the growth performance of broiler chickens supplemented with organic acids might be related to organic acid levels in the diet, the use of a single organic acid or combination of two or more organic acids, the physical and chemical properties of organic acids in acidifier products, protected or unprotected organic acids, the age or health status (challenged or not) of experimental broilers, the composition of the experimental diet, hygienic conditions of the rearing environment, etc. (Huang et al., 2024).

We further validated our growth performance results by examining intestinal pH and morphology. A longer villus is indicative of an enhanced absorptive surface area in the intestines in young chicks, whereas shorter crypt depths indicate reduced tissue turnover and a lower requirement for tissue development (Emami et al., 2012). In this study, Acidapure supplementation effectively reduced the pH of ileal contents, though it did not significantly affect the pH in the duodenum or jejunum, despite a trend towards reduction. Additionally, Acidapure supplementation improved the villus height (VH) and crypt depth (CD) ratios and the VH/CD ratio in the jejunum and ileum in the early and later stages of the experiment. This is consistent with previous studies that have confirmed that organic acids alone or in combination can improve the intestinal morphological structure of chickens (Khatun et al., 2010; Gao et al., 2021; Abou–Ashour et al., 2021) and geese (Xue et al., 2023).

The intestinal mechanical barrier is critical for maintaining mucosal function and nutrient absorption (Sánchez de Medina et al., 2014). Tight junctions, the primary intercellular connections located at the epithelial cell apex, encircle neighboring cells, blocking the passage of harmful macromolecules and microorganisms, thereby safeguarding the intestinal mucosal barrier. These junctions consist of structural proteins, including occludin, claudin, and junction adhesion molecule (JAM), and functional proteins such as ZO-1, ZO-2, ZO-3, cingulin, and zonulin (Heinemann and Schuetz, 2019). In our study, we observed that combined supplementation of Acidapure powder and liquid enhanced the expression of *Claudin-1* and *ZO-1* in the small intestine. In contrast, separate supplementation with Acidapure powder or liquid did not significantly affect gene expression, although there were numerical increases in the expression of *Occludin, Claudin-1*, and *ZO-1*. These observations align with findings by Yang et al. (2019) and Ma et al. (2021), who noted that organic acid promotes the expression of tight junction proteins in the small intestine in broilers. Additionally, organic acids have been shown to increase the expression of tight junction proteins in other species (Huang et al., 2015; Tong et al., 2016). Our results indicate that the improved growth performance of broilers associated with Acidapure supplementation may be attributed to enhancements in small intestine morphology and gut barrier function, reflected by increases in tight junction protein expression. The mechanisms underlying these results likely include improved nutrient absorption, which is suggested by the observed enhancement in villus height (VH) and crypt depth (CD) ratios in the jejunum and ileum. Longer villi increase the absorptive surface, while reduced crypt depths indicate lower tissue turnover, minimizing the energy required for maintenance of the gut epithelium and promoting nutrient utilization (Emami et al., 2012). There is increased expression of tight junction protein might have contributed to enhanced barrier function, stabilizing tight junctions, reducing gut leakage, and contributing to overall intestinal health. Organic acid blends used in the current study possibly increase endogenous gut enzyme secretion and enhance proteolytic enzyme activity, which together improve GIT passage rate and nutrient digestibility. Additionally, organic acids exhibit bacteriostatic and bactericidal effects against pathogenic bacteria, supporting overall gut health (Papatisiros et al., 2013; Khan and Iqbal, 2015).

Moreover, acidifiers in feed inhibits the growth of harmful bacteria such as Salmonella and *E. coli* by changing the pH. When the pH levels drop below 5, most pH-sensitive bacteria proliferate less; however, acid-tolerant bacteria can persist. The poultry industry faces challenges from pathogenic bacteria residing gastrointestinal tract. The gastrointestinal system serves as the first line of defense against the microbial invasion, and improving poultry performance is largely dependent on the composition of the gastrointestinal microbiota (Markovi et al., 2009). In our study, Acidapure supplementation in both powder and liquid form significantly reduced the coliform count and TVC on day 42. This is consistent with findings of Hasan et al. (2010), Ma et al. (2021) and Gao et al. (2021). The primary mechanism behind the reduction of pathogenic bacteria by acidifiers is the lowered pH in the intestine, which is less conducive to bacterial multiplication. Additionally, upon entering bacterial cells at neutral pH, acids dissociate, inhibiting bacterial cell enzymes such as decarboxylases and catalases, as well as disrupting nutrient transport systems (Huyghebaert et al., 2011). This dual mechanism supports the theory that acidifiers may selectively suppress harmful bacteria while promoting a healthier gut microflora.

The combination of acidapure liquid and powder reduced cholesterol levels, and acidapure treatment in all forms reduced triglyceride levels in broiler chickens in the current study. The cholesterol-lowering effect of dietary acidifiers has been well described in earlier studies (Kamal and Ragaa, 2014; Matty and Hassan, 2020). The cholesterol-lowering effect is attributed to the inhibition of the activity of hepatic 3-hydroxy-3-methylglutaryl coenzyme A (HMG-CoA) reductase, a key regulatory enzyme involved in cholesterol synthesis (Qureshi et al., 1988; Lee et al., 2004). Lower lipid levels may contribute to improved feed efficiency by supporting metabolic health, although further studies are warranted to clarify these effects in broilers.

Numerous studies have indicated that, broiler chicks fed with various blends of organic acids exhibit enhanced immunological response (Abbas et al. 2013; Yang et al. 2018; Scicutella et al. 2021). Organic acid supplementation has been linked to significant improvements in antibody titers against Newcastle disease (Khan and Iqbal, 2015; Abbas et al. 2013). However, in our study, we did not observe a significant effect of Acidapure on the humoral immune response against the Newcastle disease virus. This lack of effect may be attributable to variations in acid levels within Acidapure, management conditions, and the health status of the broilers.

While the study provides valuable insights, certain limitations could affect the generalizability of the findings, including variability in acid composition and formulation, differences in diet and rearing conditions, and the effects of organic acids on immune response and gut microbiota composition. The current blend of acidifiers has performed well in the non-challenge model, there is a need to test in the pathogen challenge model to ensure its efficacy. Future research could focus on identifying the most effective acid blends for various poultry diets and health conditions, incorporating microbiome profiling and immune response assessments, exploring interactions between organic acids and metabolic pathways related to growth performance, and conducting field trials under diverse environmental and management conditions to better understand their impact in varied poultry production systems. To make precise use, the acidifier doses may be adjusted based on the bird's age, and the presence of microbial load in feed and water offered to the poultry. The future of acidifier use in poultry is promising, especially as the industry pursues substitutions of antibiotics for promoting gut health, and growth performance.

## Conclusions

The present study highlights the beneficial effects of organic acids blend Acidapure both in the feed and in drinking water, on the growth performance and overall health of broilers. Our findings indicate that Acidapure supplementation leads to significant improvements in growth metrics, such as average body weight and average daily gain, alongside enhancements in intestinal morphology. Additionally, the supplementation effectively reduces total viable counts (TVC) and coliform bacteria, indicating a favorable shift in gut microbiota composition. Moreover, the combination of Acidapure powder and Acidapure liquid was shown to enhance the expression of key tight junction protein genes, specifically *Claudin-1* and *ZO-1*. This increase in gene expression is likely linked to the improved integrity of the intestinal barrier, which plays a crucial role in nutrient absorption and overall gut health. Furthermore, Acidapure supplementation resulted in significant reductions in serum cholesterol and triglyceride levels, suggesting potential metabolic benefits associated with its use.

Overall, this study provides compelling evidence that organic acid blends, such as Acidapure, hold significant promise as effective alternatives to antibiotics for enhancing gut health and improving the production performance of broilers in the post-antibiotic era. The adoption of such supplements could contribute to more sustainable poultry production practices while mitigating the risks associated with antibiotic use in animal husbandry.

## Declaration of competing interest

The authors do not have any conflict of interest for publication of the research article.
